# Food Allergy: Knowledge and Attitude of Primary School Teachers in Makkah Region, Saudi Arabia

**DOI:** 10.7759/cureus.45203

**Published:** 2023-09-14

**Authors:** Lujain Alzahrani, Hadeel H Alshareef, Hadeel F Alghamdi, Renad Melebary, Sarah N Badahdah, Razan Melebary, Mohammad Binhussein, Imad Khojah, Ameera Bukhari, Amer Khojah

**Affiliations:** 1 College of Medicine, Umm Al-Qura University, Makkah, SAU; 2 Department of Emergency Medicine, King Abdulaziz University Hospital, Jeddah, SAU; 3 College of Science, Taif University, Taif, SAU

**Keywords:** epinephrine, pediatrics, makkah region, school teachers, food allergy

## Abstract

Food allergy is a serious and potentially life-threatening medical condition that affects both adults and children. School teachers are considered to be among the first line of defense in identifying and responding to such situations, as 22% of food allergic reactions occur in schools. It is, therefore, important to understand the knowledge and attitudes of school teachers toward food allergy. This study is a descriptive, cross-sectional investigation conducted using an online questionnaire from December 2022 to February 2023. We collected data from 413 primary school teachers in Makkah region, Saudi Arabia. SPSS version 21 (IBM Corp., Armonk, NY) was used to analyze the data. Out of 413 teachers who met the inclusion criteria, only 14.5% demonstrated good awareness levels (scoring above 60% on the knowledge questionnaire) regarding food allergy, with young teachers making up the highest proportion as 26.1% of younger teachers had good awareness levels compared to 8.8% of teachers aged 51-60 years (p = 0.012). Additionally, 46.7% of the teachers knew the symptoms of a severe allergic reaction, and only 16.7% knew that they should use an epinephrine pen as the first step in managing a severe allergic reaction. School teachers have insufficient knowledge about food allergies, underscoring the importance of establishing school policies to handle food allergies. Such policies should encompass the adoption of a comprehensive food allergy action plan, training programs for school personnel, and educational campaigns.

## Introduction

Food allergy is a serious and potentially life-threatening medical condition that affects both adults and children [1]. Large electronic survey studies suggest that around 8% of children suffer from food allergies, with 3% experiencing severe reactions [2]. The term "food allergy" refers to an adverse immune response to a particular food that occurs consistently with repeated exposure [3]. While any food can potentially cause a food allergy, a small number of foods, including milk, eggs, peanuts, tree nuts, fish, shellfish, soy, and wheat, are responsible for the majority of cases [4]. Symptoms can range from mild reactions like hives to severe reactions like difficulty breathing, difficulty swallowing, and low blood pressure. Food allergies are typically due to an immunoglobulin E (IgE)-mediated reaction. Management of this condition entails avoiding allergenic food and having access to auto-injectable epinephrine, which serves as an acute intervention for anaphylactic reactions. Additionally, oral antihistamines can be used for milder allergic reactions [5]. Delayed administration of epinephrine is the leading cause of death in food allergies-induced anaphylaxis [6]. One of the main barriers to the use of auto-injectable epinephrine is the lack of knowledge about anaphylaxis and the importance of timely administration of epinephrine [7]. Other barriers include lack of access to auto-injectable epinephrine, fear of administering the medication incorrectly, a lack of confidence in using the device, and concern about side effects [7].

Food allergies can significantly impact a child's nutrition and general health, leading to dietary restrictions that can affect the child's growth and development [8,9]. Additionally, food allergies can be a significant source of stress and anxiety for both parents and children, negatively affecting their psychological well-being [10]. Since children spend a significant amount of time in the school environment, schools play a crucial role in managing food allergies. School teachers are often the first to identify and respond to allergic reactions, as 22% of such reactions occur in school, according to one study [11]. Another study found that 16% to 18% of students with food allergy experience an allergy reaction at school [12,13]. Therefore, it is crucial for school staff to be aware of food allergies and to be able to identify and manage allergic reactions in the case of an anaphylactic reaction. The objective of this study is to assess school teachers' knowledge and awareness of food allergies in the Makkah region of Saudi Arabia.

## Materials and methods

This descriptive, cross-sectional study was carried out from December 2022 to February 2023 using an online, self-administered questionnaire distributed among primary schools located in Makkah region, Saudi Arabia. Participants were recruited through various methods, such as social media apps, teachers' group lists, and phone calls. The sample size was calculated to be 379 teachers, using the single proportion equation in the OpenEpi calculator at 95% confidence intervals and a 5% accepted margin of error. The total sample size was 413 participants. The questionnaire contains the following sections: a consent form, socio-demographic data, an assessment of knowledge of food allergy, and an assessment of attitude toward food allergy. The questionnaire items were based on a previously published study by Ercan et al. [14]. We modified some answers to fit our local study population, such as the emergency service phone number. The study was approved by the Biomedical Research Ethics Committee of Umm Al-Qura University, Saudi Arabia (approval number: HAPO-02-K-012-2023-02-1474).

Data analysis

The data were analyzed using Statistical Package for Social Sciences (SPSS) version 21 (IBM Corp., Armonk, NY). The chi-square test was used to investigate the differences between categorical variables in our study. All statistical methods used were two-tailed with an alpha level of 0.05. A P-value less than or equal to 0.05 was considered significant. We assessed the overall awareness level of food allergy by summing up the scores for different correct awareness items. If the participant's score was less than 60% of the overall score, it was categorized as a poor level of awareness, and if the score was 60% or more of the overall score, it was considered a good level of knowledge. Descriptive analysis was done by prescribing frequency distribution and percentages for study variables, including teachers' personal data, medical and family history of food allergy, work data, and school preparedness for dealing with cases of food allergy. Also, awareness regarding food allergy was tabulated, while overall knowledge was graphed. Cross-tabulation for factors associated with study teachers' awareness of food allergy was carried out with a Pearson chi-square test for significance and an exact probability test if there were small frequency distributions.

## Results

Socio-demographic data

The study questionnaire was completed by 413 teachers, whose ages ranged from 21 to 60 years, with a mean age of 40.1 ± 12.7 years. Table 1 presents the demographics of the study population. Notably, 72 teachers (17.4%) had a personal history of food allergy, and 162 teachers (39.2%) had a family history of food allergy (Table 1). In terms of employment, 75.3% (311) of the teachers worked in public schools. Regarding years of teaching experience, 27.1% (112) had one to five years, 20.3% (84) had six to 10 years, and 31% (128) had more than 16 years of experience. Of teachers, 34.6% had witnessed someone suffering from a serious allergic reaction, and 12.1% had witnessed a serious allergic reaction to a student. Additionally, 35.1% knew of a student with a food allergy.

**Table 1:** Socio-demographic data of study school teachers' staff, Makkah region, Saudi Arabia

Socio-demographic data	No	%
Age in years		
21-30	69	16.7%
31-40	135	32.7%
41-50	141	34.1%
51-60	68	16.5%
Gender		
Male	136	32.9%
Female	277	67.1%
Nationality		
Saudi	385	93.2%
Non-Saudi	28	6.8%
Marital status		
Not-married	75	18.2%
Married	338	81.8%
Do you have children?		
Yes	309	91.4%
No	29	8.6%
Educational level		
Teachers Institute certificate	22	5.3%
University bachelor's degree	344	83.3%
Post-graduate degree	47	11.4%
Is there a personal history of food allergy?		
Yes	72	17.4%
No	341	82.6%
Family history of food allergy		
Yes	162	39.2%
No	251	60.8%

School teachers' awareness regarding food allergy

Table 2 shows that 46.7% of the teachers indicated knowing the symptoms of a serious allergic reaction, and 44.8% had been previously informed about anaphylaxis. The teachers reported food and drugs as the most common substances that could cause a serious allergic reaction, with 68% and 49.2%, respectively. Only 10.7% knew that exercise could cause a severe allergic reaction. Regarding specific food allergens, 44.9% of teachers reported eggs as a culprit of food allergy, 29.6% reported fish, 24.8% reported seafood, 15.5% reported nuts, 11.4% reported milk and milk products, while 9.5% reported cereals and fruits as causing serious allergies.

**Table 2:** School teachers' awareness regarding food allergies, Makkah region, Saudi Arabia

Awareness items		No	%
Do you know the symptoms of a serious allergic reaction?	Yes	193	46.7%
	No	220	53.3%
Have you been told about anaphylaxis before?	Yes	185	44.8%
	No	228	55.2%
What type of substance can cause a serious allergic reaction?	Food	281	68.0%
	Direct contact with animals	209	50.6%
	Drugs	203	49.2%
	Pollen	170	41.2%
	Mites	95	23.0%
	Cigarettes	101	24.5%
Can exercise cause serious allergies?	Yes	44	10.7%
	No	182	44.1%
	Don't know	187	45.3%
Can latex cause serious allergies?	Yes	97	23.5%
	No	64	15.5%
	Don't know	252	61.0%
Food causing serious allergy	Egg	185	44.9%
	Seafood	102	24.8%
	Nuts	64	15.5%
	Fish	122	29.6%
	Chocolate	38	9.2%
	Strawberry	19	4.6%
	Banana	75	18.2%
	Some cereals & fruits	39	9.5%
	Milk/milk products	47	11.4%
	Others	27	6.6%
	Don't know	49	11.9%
Should children with severe allergies wear something that indicates they are allergic in front of others?	Yes	248	60.0%
	No	165	40.0%
Have you ever heard of epinephrine as a treatment?	Yes	115	27.8%
	No	298	72.2%
How should epinephrine be given?	Intramuscular (IM)	44	38.3%
	Subcutaneous (SC)	18	15.7%
	Intravenous (IV)	16	13.9%
	I don't know	37	32.2%
Have you ever heard of epinephrine auto-injection?	Yes	91	22.0%
	No	322	78.0%
Do you know how to use epinephrine auto-injection?	Yes	55	21.3%
	No	203	78.7%
What is the right action to take in the event of a life-threatening allergic reaction?	Make sure the airway is open	142	34.4%
	Use an epinephrine pen	69	16.7%
	Put him/her on a hard surface	24	5.8%
	Call 997	165	40.0%
	Take a picture of the child if available	4	1.0%
	Raise his legs	9	2.2%

Approximately 60% of teachers recommended that children with severe allergies should wear something that indicates their allergy. Regarding treatment, 27.8% were familiar with epinephrine, and among those, only 38.3% knew that it should be administered via intramuscular injection. Merely 22% of teachers had heard of epinephrine auto-injection, and 21.3% knew how to use it. In the event of a life-threatening allergic reaction, only 16.7% of teachers knew that an epinephrine auto-injection should be used first, while 34.4% believed that ensuring the airway was the proper action, and 40% believed that contacting emergency services (997) was appropriate. Out of all the teachers, only a small proportion of 15% (62 teachers) were aware of an action plan to handle severe allergic reactions among students in their school. Additionally, a mere 12.6% (52 teachers) reported that their school had emergency medications on hand to be used in case of a serious allergic reaction.

**Figure 1:**
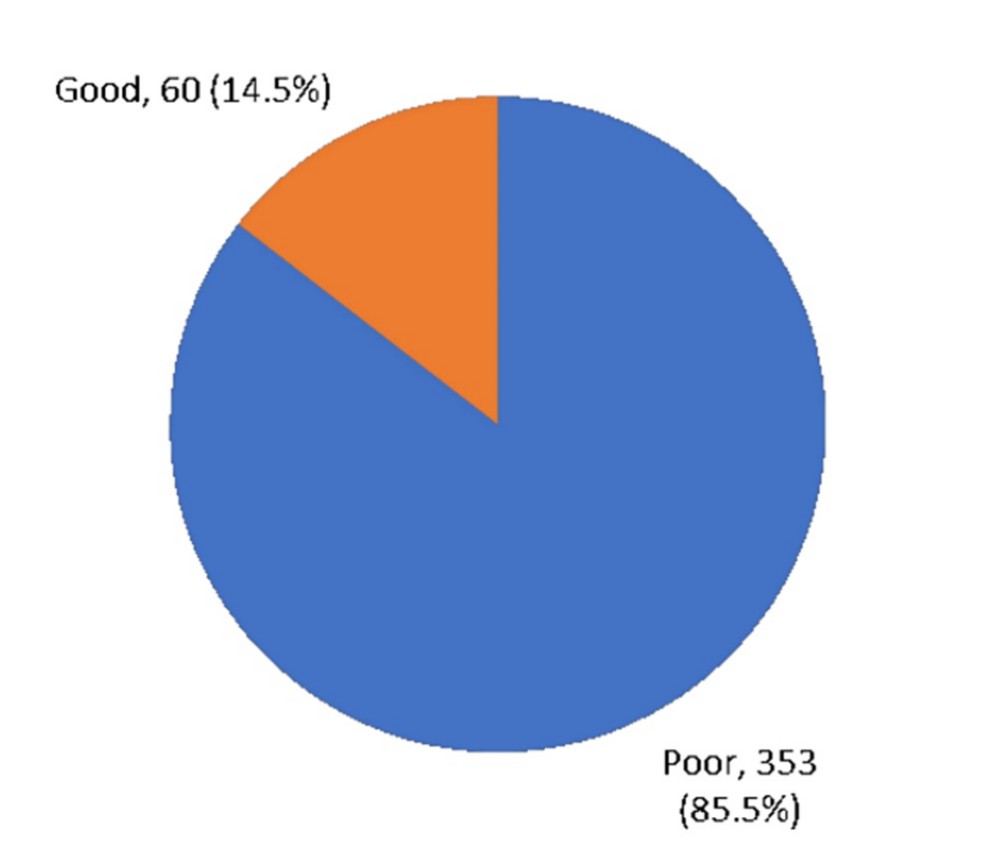
Overall teachers' awareness level regarding food allergy, Makkah region, Saudi Arabia

**Figure 2:**
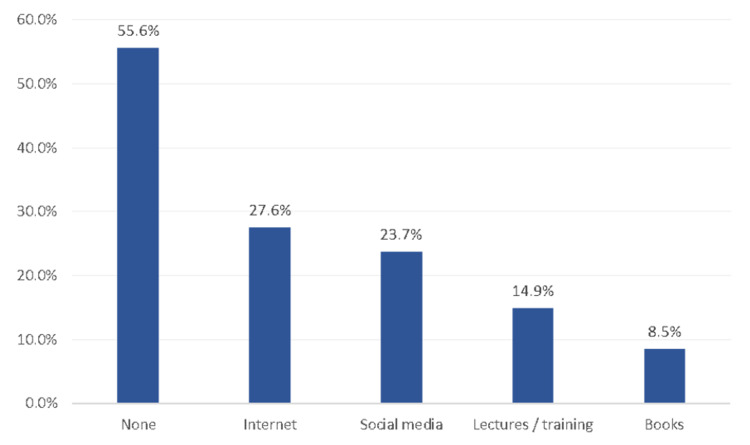
Source of teachers' information regarding food allergy, Makkah region, Saudi Arabia

Out of all the teachers, only 60 (14.5%), had a good overall understanding of food allergies, while a majority of the teachers (353, 85.5%) had a poor understanding of food allergies (Figure 1). As for the source of teachers' knowledge about food allergies, 27.6% got their information from the internet, followed by social media at 23.7%, lectures at 14.9%, and books at 8.5%. On the other hand, 55.6% of teachers did not have a specific source for the information (Figure 2).

Factors associated with teachers' awareness regarding food allergy

In relation to teachers' awareness of food allergies, Table 3 shows that younger teachers (aged below 51 years) had a significantly higher level of overall awareness with 26.1% exhibiting good awareness, compared to only 8.8% of those aged 51-60 years. Additionally, non-Saudi teachers had a better level of awareness with 39.3% showing good awareness compared to 12.7% of Saudi teachers (P = 0.001). Those with a post-graduate degree also had better awareness with 29.8% exhibiting good awareness compared to 13.6% of teachers with a lower level of education (P = 0.007). Furthermore, teachers with a personal history of allergies had a higher level of awareness with 29.2% exhibiting good awareness (P = 0.001), as did teachers in private schools (25.5%, P = 0.001). Those who had witnessed someone suffering a serious allergic reaction also had better awareness with 23.1% exhibiting good awareness (P = 0.001). Finally, teachers who obtained their information from lectures had the highest level of good awareness at 44.3% compared to only 2.6% of those who did not have a source (P = 0.001).

**Table 3:** Factors associated with teachers' awareness regarding food allergy, Makkah, Saudi Arabia P: Pearson X2 test; $: exact probability test; * P < 0.05 (significant).

Factors	Awareness level				P-value
	Poor		Good		
	No	%	No	%	
Age in years					0.012*
21-30	51	73.9%	18	26.1%	
31-40	114	84.4%	21	15.6%	
41-50	126	89.4%	15	10.6%	
51-60	62	91.2%	6	8.8%	
Gender					0.505
Male	114	83.8%	22	16.2%	
Female	239	86.3%	38	13.7%	
Nationality					0.001*^$^
Saudi	336	87.3%	49	12.7%	
Non-Saudi	17	60.7%	11	39.3%	
Do you have children?					0.221
Yes	270	87.4%	39	12.6%	
No	23	79.3%	6	20.7%	
Educational level					0.007*
Secondary	19	86.4%	3	13.6%	
Diploma/university	301	87.5%	43	12.5%	
Post-graduate degree	33	70.2%	14	29.8%	
Is there a personal history of food allergy?					0.001*
Yes	51	70.8%	21	29.2%	
No	302	88.6%	39	11.4%	
Family history of food allergy					0.118
Yes	133	82.1%	29	17.9%	
No	220	87.6%	31	12.4%	
Work school					0.001*
Governmental school	277	89.1%	34	10.9%	
Private school	76	74.5%	26	25.5%	
Experience years in teaching					0.189
1-5	90	80.4%	22	19.6%	
6-10	70	83.3%	14	16.7%	
11-15	79	88.8%	10	11.2%	
16+	114	89.1%	14	10.9%	
Have you ever had a serious allergic reaction to a student?					0.110
Yes	39	78.0%	11	22.0%	
No	314	86.5%	49	13.5%	
Have you ever seen someone suffer a serious allergic reaction?					0.001*
Yes	110	76.9%	33	23.1%	
No	243	90.0%	27	10.0%	
Source of information					0.001*
Social media	61	62.9%	36	37.1%	
Lectures/training	34	55.7%	27	44.3%	
Internet	72	63.7%	41	36.3%	
Books	20	57.1%	15	42.9%	
None	222	97.4%	6	2.6%	

## Discussion

This study aimed to assess primary school teachers' knowledge, awareness, and attitude toward food allergies in the Makkah region, Saudi Arabia. Given that approximately 22% of allergic reactions for school-age children occur in schools [11], teachers play a crucial role in responding to these reactions. Delayed use of lifesaving measures like the EpiPen can lead to severe outcomes. Our findings reveal a significant lack of knowledge among teachers, with over 80% showing poor awareness levels. This knowledge gap poses a substantial risk to children with food allergies, potentially resulting in fatal consequences [6]. Similar findings were reported in previous studies conducted in different regions of Saudi Arabia. For example, in a study conducted by Gohal [15] in Jazan, it was found that only 17.3% of school teachers had a good understanding of food allergies. The majority of teachers in that study had limited knowledge about common food allergens, such as peanuts and nuts, which are known to cause severe reactions [16]. The study found that younger, non-Saudi, private schools, and post-graduate teachers exhibited a better awareness of food allergies. Similarly, teachers with a personal history of allergies and those who witnessed someone suffering a serious allergic reaction also had better awareness.

Regarding management, the study revealed that less than 30% of the participating teachers were aware of adrenaline, and only 16.7% knew that an adrenaline pen (epinephrine) should be used in case of a life-threatening allergic reaction. A similar study conducted in Saudi Arabia in 2020 reported that 25.3% of teachers would use an antihistamine, while 16.4% would use an epinephrine injection [17]. On the other hand, a previous study conducted in New York City (NYC), USA, in 2015 found that the majority (80%) of teachers were familiar with self-injectable epinephrine [18]. This difference could be attributed to more awareness about food allergy due to the higher prevalence of food allergy in their classroom [12] or more strict laws to train teachers on using epinephrine [19]. For example, Elijah’s Law was passed in NYC recently, which mandates that childcare facilities implement measures to manage food allergies among the children in their care. These measures include developing emergency protocols, strategies for communicating food allergies to children, and plans for preventing exposure to food allergens [19].

Most studies conducted in Saudi Arabia reveal a significant lack of knowledge and awareness about food allergy [15,17], emphasizing the need for school teacher training. Given the risk associated with severe allergic reactions, policies for managing food anaphylaxis should be implemented in schools, with guidelines and training materials provided, EpiPens made available, and staff regularly retrained. For successful educational programs, collaboration between educational authorities and healthcare professionals is crucial. Workshops, seminars, and online resources can be utilized for training, making it convenient for teachers to access and engage with the materials. Alongside training, guidelines for managing food anaphylaxis should be established, outlining step-by-step procedures for teachers to follow during allergic reactions. Also, pediatricians should provide the school with individualized healthcare plans (emergency action plans) for children with food allergies. For example, a comparable educational safety campaign targeted at caregivers during childhood and adolescence resulted in a significant improvement in their understanding and attitudes toward children and adolescents' safety [20].

The study has a few noteworthy limitations. First, the reliance on self-reported data may introduce recall bias and the potential for over or underestimation of knowledge and awareness levels. Second, the study's online nature could introduce selection/recruitment bias, possibly affecting the representativeness of the sample and thereby limiting the generalizability of the findings to all school teachers. Lastly, it is important to note that the study's cross-sectional design prevents the assessment of any intervention's effectiveness, restricting our ability to directly measure the impact of specific interventions, such as education programs.

## Conclusions

This study reveals a significant lack of knowledge and awareness about food allergies among the majority of teachers in the Makkah region, Saudi Arabia. This lack of knowledge can potentially lead to serious health consequences. There is an urgent need to create educational programs and guidelines for school staff, promote anaphylaxis training, and include the proper use of auto-injectable epinephrine. Future research is required to investigate the effect of schools' policies and action plans on the level of teacher knowledge and awareness about food allergies.
